# pH-responsive micelles based on (PCL)_2_(PDEA-*b*-PPEGMA)_2_ miktoarm polymer: controlled synthesis, characterization, and application as anticancer drug carrier

**DOI:** 10.1186/1556-276X-9-243

**Published:** 2014-05-18

**Authors:** Wenjing Lin, Shuyu Nie, Di Xiong, Xindong Guo, Jufang Wang, Lijuan Zhang

**Affiliations:** 1 School of Chemistry and Chemical Engineering, South China University of Technology, Guangzhou 510640, People's Republic of China; 2 School of Bioscience and Bioengineering, South China University of Technology, Guangzhou 510640, People's Republic of China

**Keywords:** pH-responsive, Polymer, Micelles, Drug delivery, *In vitro*

## Abstract

Amphiphilic A_2_(BC)_2_ miktoarm star polymers [poly(ϵ-caprolactone)]_2_-[poly(2-(diethylamino)ethyl methacrylate)-*b*- poly(poly(ethylene glycol) methyl ether methacrylate)]_2_ [(PCL)_2_(PDEA-*b*-PPEGMA)_2_] were developed by a combination of ring opening polymerization (ROP) and continuous activators regenerated by electron transfer atom transfer radical polymerization (ARGET ATRP). The critical micelle concentration (CMC) values were extremely low (0.0024 to 0.0043 mg/mL), depending on the architecture of the polymers. The self-assembled empty and doxorubicin (DOX)-loaded micelles were spherical in morphologies, and the average sizes were about 63 and 110 nm. The release of DOX at pH 5.0 was much faster than that at pH 6.5 and pH 7.4. Moreover, DOX-loaded micelles could effectively inhibit the growth of cancer cells HepG2 with IC_50_ of 2.0 μg/mL. Intracellular uptake demonstrated that DOX was delivered into the cells effectively after the cells were incubated with DOX-loaded micelles. Therefore, the pH-sensitive (PCL)_2_(PDEA-*b*-PPEGMA)_2_ micelles could be a prospective candidate as anticancer drug carrier for hydrophobic drugs with sustained release behavior.

## Background

Over the past several decades, great efforts have been made to improve the available anticancer therapies. Unfortunately, the majority of chemotherapy, which has a substantial hydrophobic component, is usually hampered by problems such as lack of tumor selectivity, poor water solubility, uncontrollable pharmacokinetic processes, and the possible incurrence of severe side effects [1-3]. To improve therapeutic efficacy as well as minimize side effects, tremendous drug delivery vehicles based on polymer micelles have been exploited. Polymeric micelles, with nanoscopic core-shell structures self-assembled by amphiphilic copolymers, have attracted the attention of researchers as hydrophobic drug carriers owing to their unique properties, including higher loading capacity, improved water solubility, passive and active targeting capabilities, prolonged *in vivo* circulation duration, enhanced therapeutic efficacy, and negligible side effects [4-8].

In recent years, stimulus-responsive polymer materials, which can accept appropriate changes in response to specific environmental fluctuations or imposed variations of control parameters, are recognized as one of the most promising modalities in drug delivery systems due to their unique behaviors and intelligent properties [9,10]. Although many types of stimuli have been extensively studied as drug carriers, including their responsive abilities to pH, temperature, redox, light, ionic strength, enzyme and so forth, a variety of the researches have focused on utilizing pH-responsive polymeric micelles [11-15]. The vital reason for the promising use of pH-responsive polymeric micelles aiming at tumor-targeting is attributed to the different conditions in normal tissues and tumor tissues. Since the intracellular pH values of endosomal and lysosomal environment are typically acidic (pH 5.0 to 6.0 and 4.5 to 5.0, respectively) and the extracellular pH values in tumor tissues are around 6.5 to 7.0, when compared with the neutral pH 7.4 of the normal physiological environment. An ideal anticancer drug pH-responsive polymeric micelles can escape releasing of drug in normal tissues (pH 7.4) and destabilize at an early endosomal pH 6.0 [16-18]. Poly(2-(diethylamino)ethyl methacrylate) (PDEA), a kind of cationic polyelectrolyte with a p*K*_b_ of 6.9, can be soluble in water under pH 6.9 but become hydrophobic and insoluble at normal physiological conditions. The responsiveness to the weakly acidic condition indicates that PDEA copolymers can be latent pH-sensitive polymeric micelles for tumor-targeting drug delivery [16,19].

Star-shaped polymers, one kind of dendritic polymers with well-defined architecture and multiple polymer chains emanating from the central core, have similar topological structures to polymeric micelles and can form more stable nanoscale assemblies in selective solvents, as compared with the corresponding linear block analogues. Hence, star polymers have been actively investigated currently for potential utility as nanoreactors, catalysts, sensors, polymer and electrolytes and in biomedical and therapeutic applications [20-23]. Amphiphilic star polymer can be divided into amphiphilic homo-arm star block polymer (AB)_n_ and amphiphilic miktoarm star polymers (A_m_B_n_). With same polymer chains emanating from the central core, amphiphilic homo-arm star block polymers have been prepared and used particularly in drug and gene delivery [24,25]. For example, He and coworkers synthesized well-defined four-arm PEO-*b*-PDEAEMA, which could form pH-responsive micelles. And the four-arm PEO-*b*-PDEAEMA micelles were suggested high gene transfection efficiency for the delivery of DNA [26,27]. Knop's group developed amphiphilic star-shaped block copolymers (PCL_a_-*b*-POEGMA_b_)_4_ for loading the novel fungicide to provoke an inhibition of the growth of different fungal strains [28]. A series of amphiphilic four- and six-armed star triblock copolymers 4/6AS-PCL-*b*-PDEAEMA-*b*-PPEGMA were also developed recently by our group for the intracellular delivery of the anticancer drug doxorubicin (DOX) [29].

Amphiphilic miktoarm star polymers with at least two different polymer chains emanating from the central core such as A_2_B_2_, A_3_B_3_, A_2_B, A_3_B, ABC, AB_2_C_2_, ABCD, etc., especially for A_2_B_2_ and A_3_B_3_, have been used in self-assembly and responsive behavior. Gou's group synthesized a series of A_2_B_2_ miktoarm star copolymer C4S(PCL)_2_-(PEG)_2_, which could self-assemble into various morphologies in aqueous solution controlled by both the macromolecular architectures and the compositions of the copolymer [30]. Well-defined (PNIPAAM)_2_-(PNVP-*b*-PAA)_2_ and (PNIPAAM-*b*-PAA)_2_-(PNVP)_2_ were developed by Zhang's group, and by tuning pH values and temperatures of aqueous solution of these two copolymers, three types of micellar aggregates and the unimer state could undergo reversible switch on and off in size and morphology [31]. However, limited work of A_2_B_2_ and A_3_B_3_ type miktoarm polymers was reported on drug and gene delivery.

**Figure 1:**
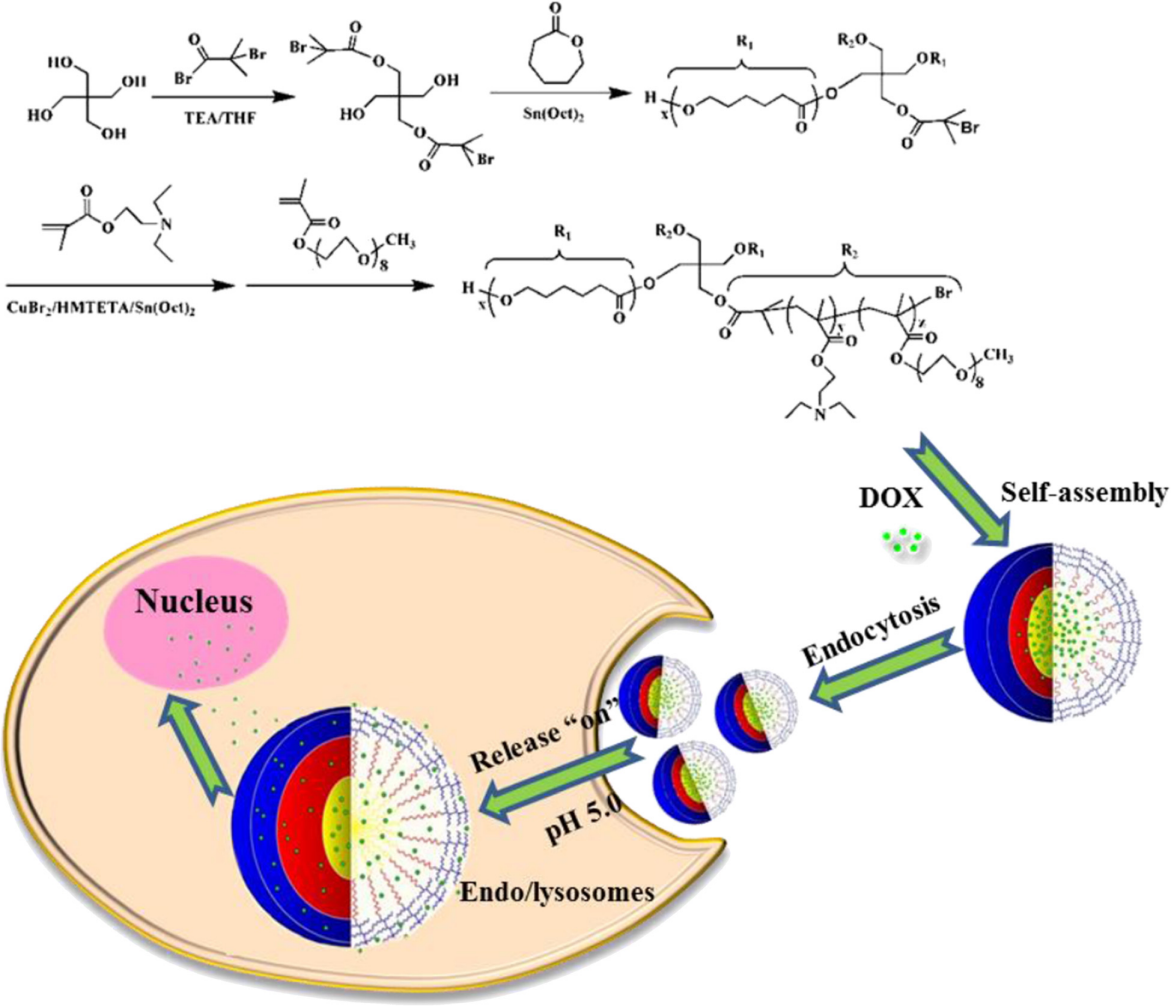
**Illustration of DOX-loaded (PCL)**_**2**_**(PDEA- *b *-PPEGMA)**_**2**_** micelles formation and intracellular DOX delivery triggered by endosomal pH (pH 5.0).**

In the current work, we report on the fabrication of amphiphilic A_2_(BC)_2_ miktoarm poly(ϵ-caprolactone)_2_-[poly(2-(diethylamino)ethyl methacrylate)-*b*-poly(poly (ethylene glycol) methyl ether methacrylate)]_2_ [(PCL)_2_(PDEA-*b*-PPEGMA)_2_] polymeric micelles as an integrated platform for intracellular delivery of the anticancer drug doxorubicin (Figure 1). Miktoarm star polymers (PCL)_2_(PDEA-*b*-PPEGMA)_2_ were synthesized by using the difunctional initiator for sequential ring opening polymerization (ROP) of ϵ-CL and continuous activators regenerated by electron transfer atom transfer radical polymerization (ARGET ATRP) of DEA and PEGMA. In aqueous solution, (PCL)_2_(PDEA-*b*-PPEGMA)_2_ could exist as structurally stable micelles possessing a hydrophobic PCL inner core, a pH-sensitive PDEA middle layer, and a hydrophilic PPEGMA outer shell. The pH-responsive PDEA layer is hydrophobic and collapses on the core at the physiological pH (7.4) which can prevent the premature burst drug release, but it becomes highly positively charged by protonation of the pendant tertiary amine groups and could lead the micelles to be adsorbed onto negatively charged cell membranes and subsequently endocytosed by tumor cells at tumor extracellular pH. Once internalized and transferred to a lysosome, the further charged PDEA can lead to faster release of the entrapped drug into the cytoplasm and nucleus [16]. Anti-tumor activities and intracellular uptake of drug-loaded (PCL)_2_(PDEA-*b*-PPEGMA)_2_ micelles were also investigated.

## Methods

### Materials

Pentaerythritol was dried under reduced pressure overnight prior to use. ϵ-Caprolactone (ϵ-CL, 99%, Aldrich, St. Louis, MO, USA) was dried over calcium hydride and distilled under reduced pressure before use. 2-(Diethylamino)ethyl methacrylate (DEA, TCI-EP) was distilled from calcium hydride and stored under argon at −20°C. Poly(ethylene glycol) methyl ether methacrylate (PEGMA, *M*_n_ = 475 Da, 99%, Aldrich) was purified by passing through a column filled with neutral alumina to remove inhibitor. Tetrahydrofuran (THF) was dried over sodium using benzophenone as a dryness indicator and distilled under nitrogen prior to use. Toluene was distilled from calcium hydride. Doxorubicin hydrochloride (DOX∙HCl) was purchased from Beijing Huafeng United Technology Co., Ltd., Beijing, China. Dulbecco's modified Eagle medium (DMEM), fetal bovine serum (FBS), penicillin, and streptomycin were all purchased from Invitrogen, Carlsbad, CA, USA. HepG2 cells were purchased from the American Type Culture Collection (ATCC), Manassas, VA, USA, and cultured under the recommended conditions according to the supplier. 3-(4,5-Dimethyltlliazol-2-yl)-2,5-diphenyltetrazoxium bromide (MTT) and Hoechst 33324 were purchased from Sigma Chemical Co. Pyrene (99%, Aldrich), 2-bromoisobutyryl bromide (98%, Alfa Aesar, Ward Hill, MA, USA), 1,1,4,7,10,10-hexamethyltriethylenetetramine (HMTETA, 99%, Aldrich), paraformaldehyde (99%, Aldrich), CuBr_2_, methanol, stannous octoate (Sn(Oct)_2_), triethylamine (TEA), dimethyl sulfoxide (DMSO), acetone, and all other reagents were used as received.

### Synthesis of difunctional initiator pentaerythritol bis(2-bromoisobutyrate) [(OH)_2_-Br_2_]

(OH)_2_-Br_2_ was synthesized as follows: to a flame-dried 250 mL Schlenk flask with a magnetic stirring bar, which was evacuated and flushed with argon thrice, pentaerythritol (6.80 g, 0.05 mmol), anhydrous THF (150 mL), and TEA (13.89 mL, 0.10 mmol) were added in turn at 0°C. Then, 2-bromoisobutyryl bromide (12.36 mL, 0.10 mmol) was injected dropwise for a period of 2 h with vigorous stirring. The reaction was continued at 0°C for 5 h and then at room temperature for another 24 h. The reaction mixture was cooled, extracted with 300 mL diethyl ether thrice, and then the diethyl ether layer was washed successively with water, saturated NaHCO_3_, and water and dried over MgSO_4_ overnight followed by rotary evaporation to remove the solvent. The colorless liquid product (OH)_2_-Br_2_ was collected by distillation under reduced pressure. ^1^H NMR (*d*_6_-DMSO as solvent, in Additional file 1: Figure S1): −O-CH_2_- *δ* = 3.65 ppm (4H), −COO-CH_2_- *δ* = 4.31 ppm (4H), −C(CH_3_)_2_-Br *δ* = 1.96 ppm (12H); Element Analysis, calculated (%): C 35.94, H 5.37; found (%): C 35.83, H 4.85.

### Synthesis of bromide-terminated two-arm poly(ϵ-caprolactone) macroinitiator [(PCL)_2_-Br_2_]

(PCL)_2_-Br_2_ was synthesized by ROP of ϵ-CL using (OH)_2_-Br_2_ as initiator [32,33]. Typically, a flame-dried 100 mL Schlenk flask equipped with a magnetic stirring bar was charged with difunctional initiator [(OH)_2_-Br_2_] (0.434 g, 1 mmol), and the flask was evacuated and flushed with argon three times. Subsequently, the freshly distilled ϵ-CL (6 g) and a required amount of Sn(Oct)_2_ (0.1 wt.% of ϵ-CL, 0.006 g) solution were injected into the flask by syringe and three ‘freeze-pump-thaw’ cycles were performed to remove any oxygen from the solution. The flask was immersed into a thermostated oil bath at 130°C for 24 h. The crude polymer was dissolved in approximately 50 mL THF followed by adding dropwise to 500 mL water/methanol (1:1, *v*/*v*) mixture to precipitate the product, which was collected and dried under vacuum for 24 h, resulting in powdery (PCL)_2_-Br_2_.

### Synthesis of A_2_(BC)_2_ miktoarm star polymers (PCL)_2_(PDEA-b-PPEGMA)_2_

The continuous ARGET ATRP of DEA and PEGMA was *in situ* monitored by ReactIR iC10 (Metter-Toledo AutoChem, Columbia, MD, USA) equipped with a light conduit and DiComp (diamond composite) insertion probe [34,35]. The FTIR spectra were collected every minute, and the change of absorbance at 938 cm^−1^ (=CH_2_ wags of the DEA and PEGMA) was used to provide the conversion of monomers during the polymerization calculated by ReactIR 4.1 software. In a typical synthesis procedure, a previously dried 100 mL Schlenk flask equipped with a magnetic stirring bar was charged with (PCL)_2_-Br_2_ (4.0 g, 0.8 mmol) and CuBr_2_ (0.0143 g, 0.064 mmol). The real-time FTIR probe was introduced into the flask, and the flask was then evacuated and flushed with argon thrice. Anhydrous toluene (18 mL), DEA (4.8 g), and ligand HMTETA (0.164 mL, 0.64 mmol) were injected into the flask using degassed syringes in order. The mixture was stirred for 10 min, and a required amount of Sn(Oct)_2_ (0.259 g, 0.64 mmol) solution in toluene (2 mL) was added into the flask by syringe. The flask was placed in a preheated oil bath maintained at 70°C, and the FTIR spectra were collected at the time. After 5 h, the absorbance of 938 cm^−1^ was kept almost constant and the second monomer PEGMA (*M*_n_ = 475, 6.4 g) was then introduced by syringe to continue the polymerization for another 20 h. Then, the flask was removed from the oil bath and cooled to room temperature. THF (50 mL) was added into the flask, and the mixture was then passed through a neutral alumina column to remove the catalyst. After removing the catalyst, the product was recovered by being precipitated into tenfold excess of *n*-hexane, filtered, and finally dried under vacuum for 24 h.

### CMC measurement

The critical micelle concentration (CMC) values of (PCL)_2_(PDEA-*b*-PPEGMA)_2_ were determined by the fluorescence probe technique using pyrene as a fluorescence probe. Pyrene dissolved in acetone was added into deionized water (pH 7.4) to make a concentration of 12 × 10^−7^ M following by removed acetone 2 h through evaporation. The final concentration of pyrene was adjusted to 6 × 10^−7^ M. The (PCL)_2_-(PDEA-*b*-PPEGMA)_2_ (5 mg) was first dissolved into 50 mL deionized water and then diluted to a series of concentrations from 0.0001 to 0.1 mg/mL with deionized water. Then, 10 mL of polymer solutions at different concentrations were added to the pyrene-filmed vials, respectively, and the combined solutions were equilibrated at room temperature in the dark for 24 h before measurement. The fluorescence excitation spectra of polymer/pyrene solutions were measured and used for determining the CMC values.

### Preparation of empty and DOX-loaded micelles

The empty and DOX-loaded (PCL)_2_(PDEA-*b*-PPEGMA)_2_ self-assembled micelles were prepared according to the diafiltration method. Typically, (PCL)_2_(PDEA-*b*-PPEGMA)_2_ (40 mg) was dissolved in 20 mL of DMSO (40 mL for empty micelles) at room temperature 25°C, followed by adding a predetermined amount of DOX∙HCl (10 mg) and double molar amount of TEA in another 20 mL of DMSO and then stirring for 4 h. Then, the mixture solution was transferred to dialysis bag (MWCO = 3.5 kDa) and dialyzed against deionized water for 24 h to remove the organic solvents and free DOX. The deionized water was changed every 4 h for the first 8 h and then replaced every 6 h. After dialysis, the micelles were filtered by a membrane filter (0.45-μm pore) to remove aggregated particles. Then, half of the empty and DOX-loaded micelles were used to study the pH-responsive behavior by the addition of NaOH or HCl (0.01 M) solution. And the remaining empty and DOX-loaded micelles were collected by freeze-drying to obtain dried product. The received white powder was stored at −20°C until further experiments. The values of *D*_h_s and morphologies of the empty and DOX-loaded micelles were monitored by DLS and TEM. DOX-loaded micelles were dissolved in 10 mL of DMSO under vigorous vortexing and analyzed by UV-vis spectrophotometer (UV-2450, Shimadzu, Kyoto, Japan) at 480 nm to obtain DOX loading content (LC), wherein a calibration curve was obtained with DOX-DMSO solutions with different DOX concentrations. The LC values were around 10% in the current work.

### *In vitro* DOX release

The release profiles of DOX from the DOX-loaded micelles at a concentration of 1 mg/mL were studied in different media (pH 5.0, pH 6.5, and pH 7.4). Briefly, 5 mg of DOX-loaded micelles were immersed in 5 mL of PBS buffer (pH 7.4 or pH 6.5) or acetate buffer (pH 5.0) and then placed in a pre-swollen cellulose membrane bag (MWCO = 3.5 kDa). The whole bag was placed into 40 mL of PBS or acetate buffer with constant shaking (100 rpm) at 37°C (Dissolution Tester RCZ-8B, TDTF, Tianjin, China). At predetermined time intervals, a 4-mL buffer solution outside the dialysis bag was extracted and it was replaced by an equal volume of fresh media to keep the sink condition. The amounts of released DOX in different buffers were monitored by UV-vis spectrophotometer at 480 nm. Each experiment was done in triplicate, and the results were the average data.

### Cell culture and cytotoxicity assay

The *in vitro* cytotoxicity tests of the free DOX, empty, and DOX-loaded micelles were evaluated by the standard MTT assay against HepG2 cells. The HepG2 cells were first seeded on a 96-well plate at an initial density of 1 × 10^4^ cells/well in DMEM supplemented with 10% FBS, penicillin (100 units/mL), and streptomycin (100 μg/mL) at 37°C in a CO_2_ (5%) incubator for 3 days to reach 60% to 70% confluence. Then, the empty micelles with the final concentration from 1 to 400 μg/mL were added. After 48 h, 20 μL of MTT solution (5 mg/mL in PBS buffer) was added into each well and incubated for another 4 h. Afterwards, the medium in each well was then removed and 200 μL of DMSO was added to dissolve the internalized purple formazan crystals. The absorbance was measured at a wavelength of 490 nm by a microplate reader (Multiskan Spectrum, Thermo Scientific, Vantaa, Finland). Data were expressed as average ± SD (*n* = 3).

HepG2 cells were incubated with free DOX and DOX-loaded micelles with DOX final concentration from 0.1 to 20 μg/mL in culture medium. After 24 and 48 h, 20 μL of MTT solution (5 mg/mL in PBS buffer) was added into each well and incubated for another 4 h. Afterwards, the culture medium was removed, the obtained crystals were dissolved in 200 μL of DMSO, and the absorbance was measured at a wavelength of 490 nm by a microplate reader. Data were expressed as average ± SD (*n* = 3).

### CLSM observation

Confocal laser scanning microscopy (CLSM, Zeiss, LSM 510, Oberkochen, Germany) was employed to examine the intracellular distribution of DOX. HepG2 cells were seeded on slides on a 6-well plate at a density of 4 × 10^5^ cells/well in 2 mL of DMEM and were cultured for 24 h at 37°C in 5% CO_2_ atmosphere. The cells were then incubated with free DOX and DOX-loaded micelles at a final DOX concentration of 50 μg/mL in DMEM for 4 or 24 h at 37°C. At each predetermined time, the culture media were removed and the cells were washed with PBS (1 min × 3) to remove the DOX-loaded micelles that were not ingested by the cells. Subsequently, the cells were fixed with 4% (*w*/*v*) paraformaldehyde aqueous solution for 30 min at room temperature. The slides were then rinsed with PBS (2 min × 3). Finally, the cells were stained with Hoechst 33324 (5 mg/mL in PBS) at 37°C for 15 min, and the slides were rinsed with PBS (2 min × 3). The prepared slides were obtained by CLSM.

### Characterization

^1^H NMR spectra measurements were examined in *d*_6_-DMSO and CDCl_3_ at 25°C using Bruker AVANCE ΙΙΙ 400 (Madison, WI, USA) operating at 400 MHz. The number average molecular weight (*M*_n_) and polydispersity index (*M*_w_/*M*_n_) were determined by gel permeation chromatography (GPC) adopting an Agilent 1200 series GPC system (Santa Clara, CA, USA) equipped with a LC quant pump, PL gel 5 mm 500, 10^4^, and 10^5^ Å columns in series, and RI detector. The column system was calibrated with a set of monodisperse polystyrene standards using HPLC grade THF as mobile phase with a flow rate of 1.0 mL/min at 30°C. Fluorescence spectra were recorded using a fluorescence spectrophotometer (F-4500, Hitachi, Chiyoda-ku, Japan). The hydrodynamic diameter (*D*_h_) and distribution (PDI) of micelles were measured by dynamic light scattering (DLS, Malvern Zetasizer Nano S, Malvern, WR, UK). Morphologies of micelles were investigated by transmission electron microscopy (TEM, Hitachi H-7650) operating at 80 kV.

## Results and discussion

### Synthesis and characterization of (PCL)_2_(PDEA-*b*-PPEGMA)_2_

**Figure 2:**
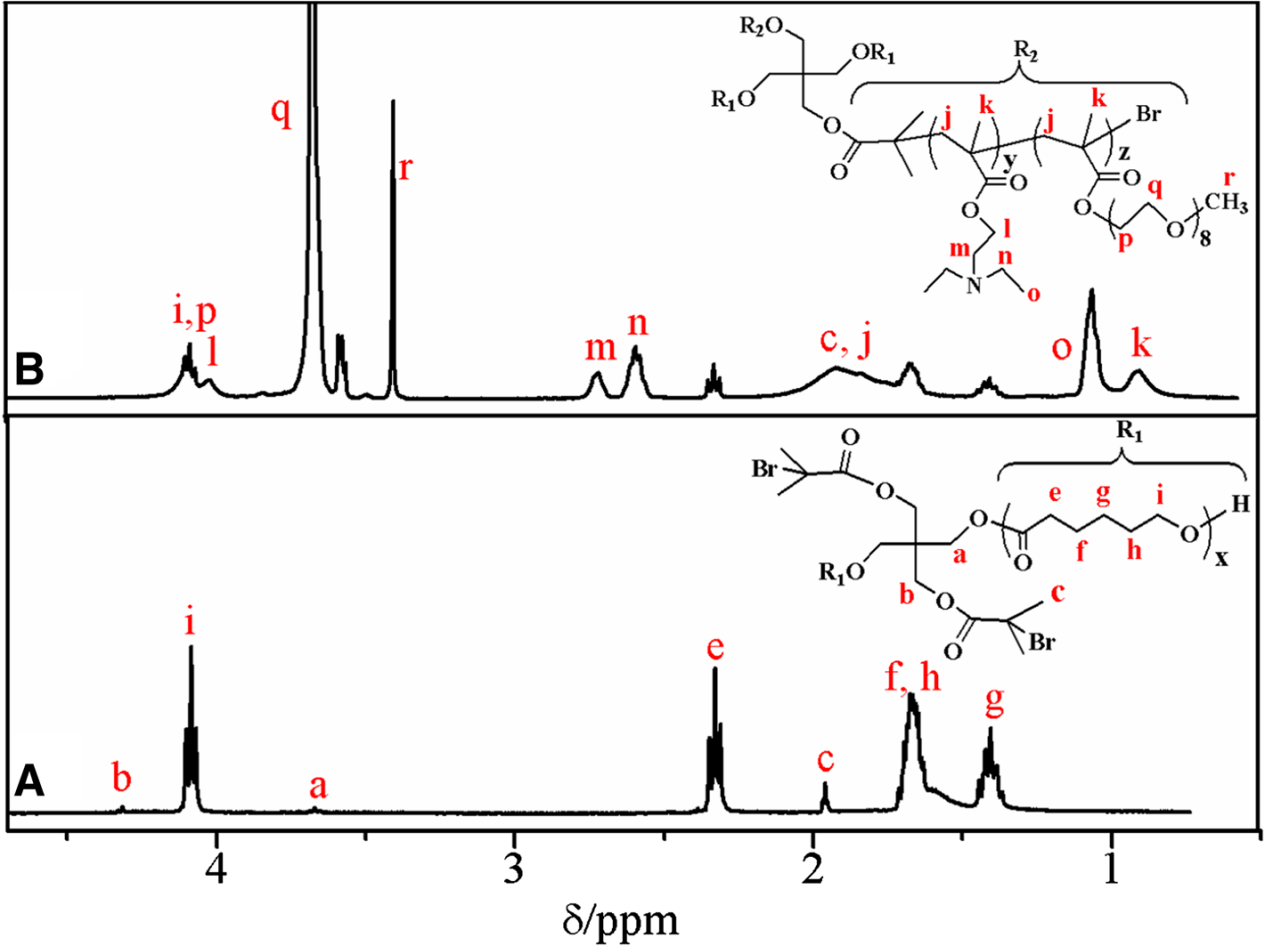
^**1**^**H NMR spectra of (PCL)**_**2**_**-Br**_**2 **_**(A) and (PCL)**_**2**_**(PDEA- *b *-PPEGMA)**_**2 **_**(B) in CDCl**_**3**_**.**

A_2_(BC)_2_ miktoarm star polymers (PCL)_2_(PDEA-*b*-PPEGMA)_2_ were synthesized by using the difunctional initiator for sequential ROP of ϵ-CL and continuous ARGET ATRP of DEA and PEGMA, as illustrated in Figure 1. Representative ^1^H NMR spectra of (PCL)_2_-Br_2_ and (PCL)_2_(PDEA-*b*-PPEGMA)_2_ were depicted in Figure 2, and all of the peaks corresponding to characteristic hydrogen atoms were labeled. In Figure 2A, the characteristic signals at 1.96, 3.65, and 4.31 ppm were assigned, respectively, to -C(CH_3_)_2_-Br, −O-CH_2_-, and -COO-CH_2_- in the pentaerythritol unit, whereas the characteristic signals at 1.40, 1.66, 2.33, and 4.10 ppm were from -CH_2_- protons of PCL backbone. In Figure 2B, the signals at 0.90 and 1.82 to 1.92 ppm are assigned respectively to -CCH_3_ and -CH_2_- of methacrylate backbone. The signals at 2.71 and 4.01 ppm were the characteristic resonances of the coterminous two methylene protons of -CH_2_CH_2_- in DEA unit, and the signals at 1.05 and 2.59 ppm belonged respectively to the end methyl and methylene protons of -CH_2_CH_3_ in DEA unit. The characteristic PEGMA peaks at 3.40, 3.65, and 4.35 ppm attributed to -OCH_3_, −OCH_2_-CH_2_O-, and -COO-CH_2_- protons, respectively. The degree of polymerization of PCL (*x*), PDEA (*y*) and PPEGMA (*z*) and the molecular weights (*M*_n,NMR_) were calculated from the integration ratio values of signal (g) to (a) (*I*_g_/*I*_a_), signal (n) to (g) (*I*_n_/*I*_g_), and signal (r) to (g) (*I*_r_/*I*_g_), respectively, as summarized in Table 1.

**Table 1:** **GPC and**^**1**^**H NMR data of (PCL)**_**2**_**(PDEA- *b *-PPEGMA)**_**2**_**polymers**

**Entry**	**Sample**^**a**^	***M***_**n, GPC**_^**b**^	***M***_**w**_**/*M***_**n**_^**b**^	***M***_**n, NMR**_^**c**^	***M***_**n, RealIR**_^**d**^
1	(PCL_24_)_2_(PDEA_16_-*b*-PPEGMA_19_)_2_	14,888	1.28	29,617	28,200
2	(PCL_24_)_2_(PDEA_37_-*b*-PPEGMA_15_)_2_	12,692	1.19	33,977	34,300
3	(PCL_38_)_2_(PDEA_26_-*b*-PPEGMA_11_)_2_	18,302	1.19	29,530	28,524
4	(PCL_38_)_2_(PDEA_17_-*b*-PPEGMA_9_)_2_	13,586	1.35	24,480	24,614
5	(PCL_32_)_2_(PDEA_25_-*b*-PPEGMA_22_)_2_	19,389	1.41	37,766	38,114
6	(PCL_32_)_2_(PDEA_20_-*b*-PPEGMA_19_)_2_	18,707	1.37	32,907	32,120

^a^The subscripts of PCL, PDEA and PPEGMA were the DP of PCL (x), PDEA (*y*) and PPEGMA (*z*) calculated from ^1^H NMR spectrum; ^b^measured by GPC in THF^; c^calculated by the equations *M*_n, NMR_ = (114 × *x +*185 × *y +* 475 × *z* ) × 2 + 434^; d^calculated by monomer conversion from the ReactIR.

**Figure 3:**
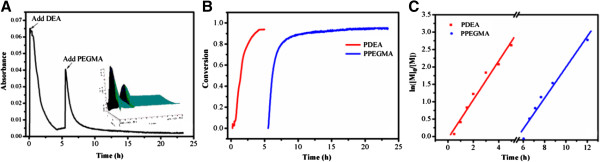
***In situ *monitored by ReactIR iC10.** The absorbance at 938 cm^−1^ (=CH_2_ wags) **(A)** monomer conversion versus time curves **(B)** and kinetic plots **(C)** for continuous ARGET ATRP of DEA and PEGMA.

Figure 3 showed that the reaction process could be easily *in situ* monitored by ReactIR iC10 via detecting the change of absorbance at 938 cm^−1^ (=CH_2_ wags of the DEA and PEGMA) [36,37]. It could be seen that the absorbance at 938 cm^−1^ decreased as the polymerization of DEA proceeded. Since the absorbance of DEA almost kept constant at 5 h, the second monomer PEGMA was added to continue the polymerization for another 20 h until the absorbance remained unchanged again in Figure 3A. From the change of absorbance at 938 cm^−1^*in situ* monitored by react infrared spectroscopy, we could calculate the conversions of DEA and PEGMA during the ARGET ATRP presented in Figure 3B. And thus the molecular weights (*M*_n, ReactIR_) of the (PCL)_2_(PDEA-*b*-PPEGMA)_2_ could be calculated from the conversions of DEA and PEGMA, which was seldom reported before. The *M*_n, ReactIR_ listed in Table 1 were in good agreement with the *M*_n,NMR_, suggesting that (PCL)_2_(PDEA-*b*-PPEGMA)_2_ with different PCL/PDEA/PPEGMA contents were well-defined. The semilogarithmic plots of ln([M]_o_/[M]) vs. time from Figure 3C showed linear time dependency for both DEA and PEGMA during their polymerization, indicating that a good control of the polymerization process was achieved in the current work.

The molecular weights of the serial (PCL)_2_(PDEA-*b*-PPEGMA)_2_ were determined by GPC and summarized in Table 1. It can be seen that the GPC curves presented in Additional file 1: Figure S2 appeared monomodal symmetric distribution and the values of *M*_w_*/M*_n_ were below 1.50, which are acceptable for further application of delivering drugs. It was also found that GPC analysis for (PCL)_2_(PDEA-*b*-PPEGMA)_2_ tended to underestimate the molecular weight (which was typically smaller) as compared to their linear counterpart due to the reduced hydrodynamic volumes. The characterization of the molar masses of star polymers by GPC is not straightforward. Since standard samples with exactly the same topology and with known molar masses do not exist, the calibration with narrow standards cannot be applied [38,39].

### Characterization of the empty and DOX-loaded micelles

**Figure 4:**
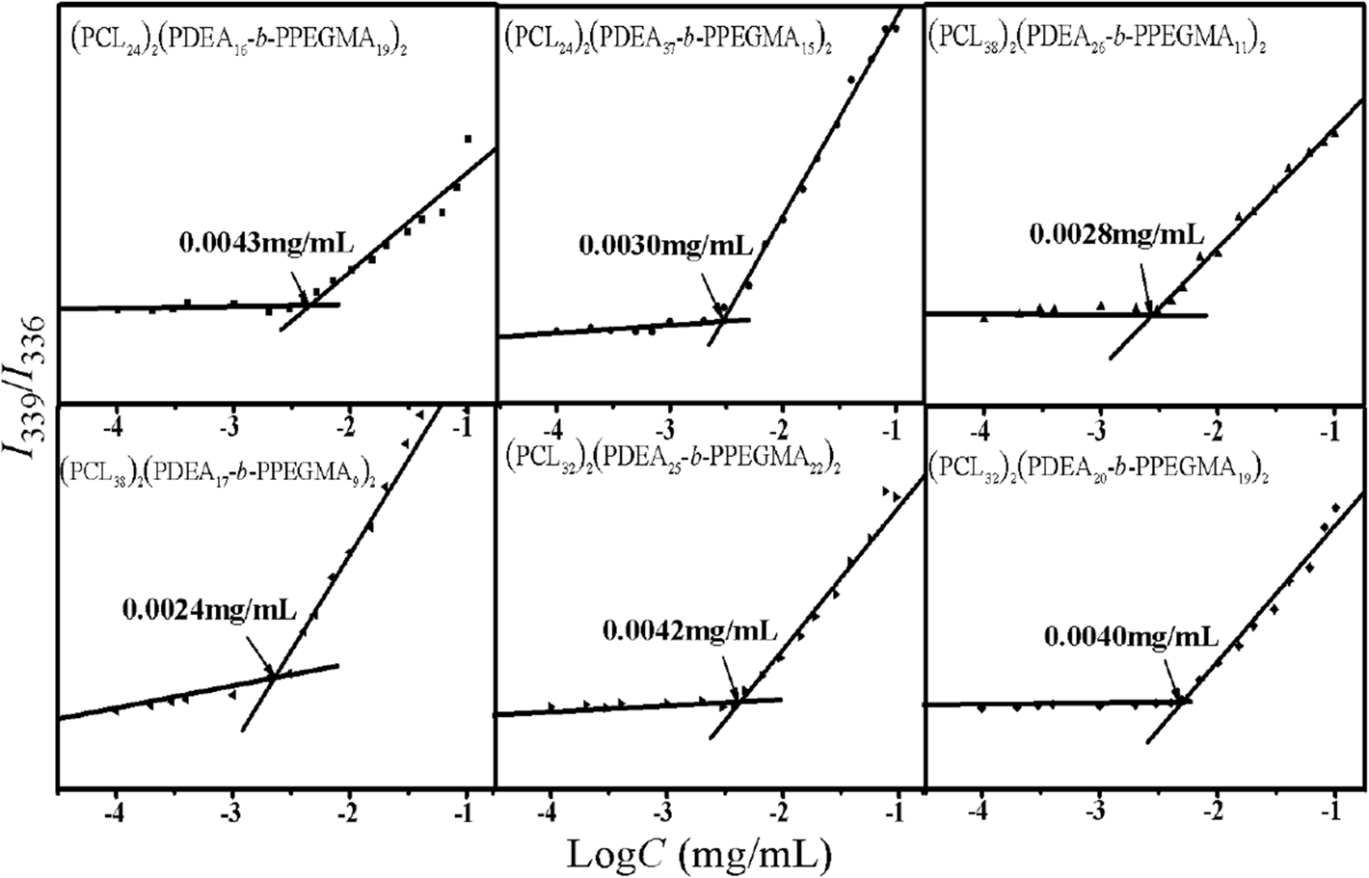
**Graphs of intensity ratios (*I***_**339**_**/*I***_**336**_**) as function of logarithm of (PCL)**_**2**_**(PDEA- *b *-PPEGMA)**_**2**_** concentrations in aqueous solution.**

The formation of micelles self-assembled from (PCL)_2_(PDEA-*b*-PPEGMA)_2_ in aqueous phase was verified using a fluorescence technique with pyrene as a fluorescence probe [40-42]. When the (PCL)_2_(PDEA-*b*-PPEGMA)_2_ micelles were formed, pyrene molecules preferably located inside or closed to the hydrophobic core of micelles, and consequently, the photophysical characteristics were changed. In the excitation spectra of polymer/pyrene solutions (see Additional file 1: Figure S3), with increasing the concentrations of (PCL)_2_(PDEA-*b*-PPEGMA)_2_, the fluorescence intensity increased and the (0, 0) band shifted from 336 to 339 nm in the excitation spectra of pyrene. The ratios of *I*_339_ to *I*_336_ were plotted against (PCL)_2_(PDEA-*b*-PPEGMA)_2_ concentrations, which can be seen in Figure 4. The CMC values of (PCL)_2_(PDEA-*b*-PPEGMA)_2_ were determined from the crossover points which were in the range of 0.0024 to 0.0043 mg/mL, increasing as the weight fraction of PCL decreased [43]. For example, the CMC values 0.0043, 0.0040, and 0.0024 mg/mL of (PCL_24_)_2_(PDEA_16_-*b*-PPEGMA_19_)_2_, (PCL_32_)_2_(PDEA_20_-*b*-PPEGMA_19_)_2_, and (PCL_38_)_2_(PDEA_17_-*b*-PPEGMA_9_)_2_, respectively, were decreased in order. Moreover, as the samples were prepared with deionized water (pH 7.4), most tertiary amine residues of PDEA were still deprotonated and exhibited as hydrophobic. Hence, taken the hydrophobicity of PDEA block into the consideration, the CMC of (PCL_24_)_2_(PDEA_37_-*b*-PPEGMA_15_)_2_ (0.0030 mg/mL) was much lower than the CMC of (PCL_24_)_2_(PDEA_16_-*b*-PPEGMA_19_)_2_ (0.0043 mg/mL).

**Figure 5:**
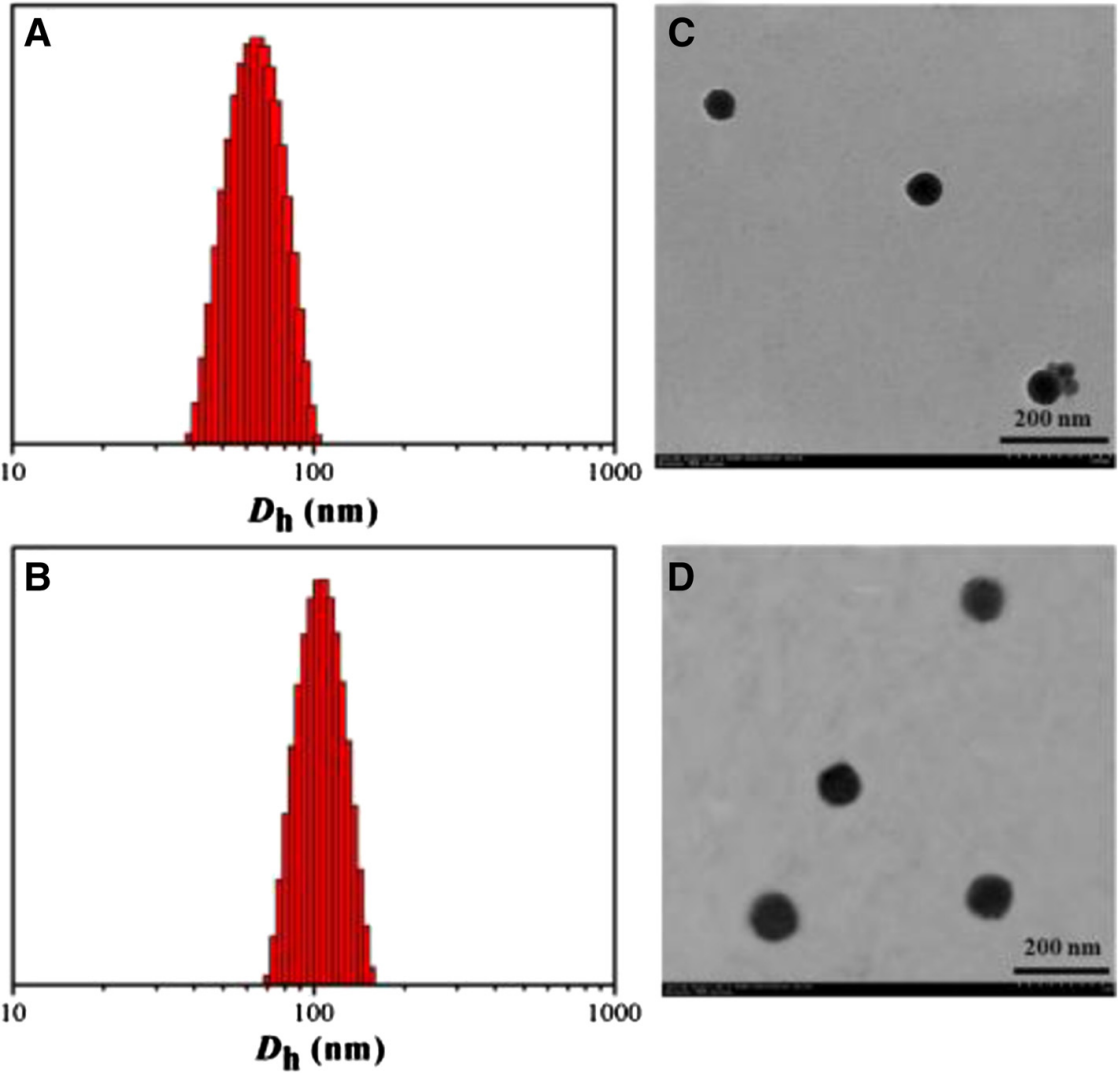
Size distribution determined with DLS (A,B) and TEM (C,D) for empty micelles (A,C) and DOX-loaded micelles (B,D).

**Figure 6:**
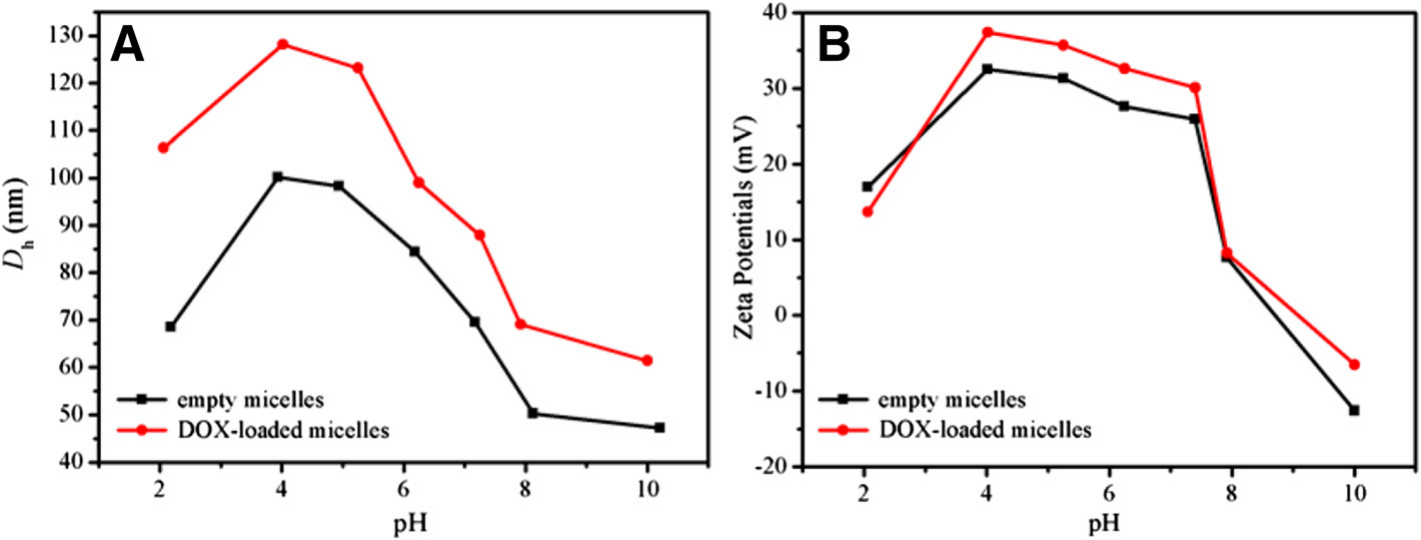
***D***_**h**_** (A) and zeta potential (B) results of empty micelles and DOX-loaded micelles at different pH.**

The (PCL_24_)_2_(PDEA_16_-*b*-PPEGMA_19_)_2_ was used as an example to encapsulate hydrophobic drug DOX. The *D*_h_ of the empty micelles self-assembled from the polymer (PCL_24_)_2_(PDEA_16_-*b*-PPEGMA_19_)_2_ at pH 7.4 was 63 nm observed by DLS measurement. After drug loading, the DOX-loaded micelles showed a larger size than the empty micelles with *D*_h_s around 110 nm, which were shown in Figure 5A,B. It was because hydrophobic DOX promoted hydrophobic interaction among the PCL chains and thus led to an increase in aggregation. The micellar size maintained narrow unimodal distribution, indicating good physical performance of the assembled micelles. Figure 5C,D showed the TEM images of empty micelles, and DOX-loaded micelles were spherical in shape (pH 7.4). It is worthwhile to note that the average sizes shown in TEM images were almost in accordance with the DLS results. The empty and DOX-loaded micelles possessed positive charges in pH 7.4 due to the pendant tertiary amine groups in the PDEA chains (Figure 6B). The highly charged character of the (PCL)_2_(PDEA-*b*-PPEGMA)_2_ micelles can prevent the aggregation of micelles, extend blood circulation times, increase the interactions between micelles and cell membranes which can facilitate penetrating of cell membranes [44,45].

The variations of the *D*_h_s and zeta potentials of the empty micelles and DOX-loaded micelles were investigated from the facile pH adjusting. As shown in Figure 6, when decreasing pH from 10 to 2, the *D*_h_s and zeta potentials increased gradually followed by abrupt descend because the micelles underwent shrinking-swelling-dissociating conformational transition. The *D*_h_s of the micelles showed slightly increase owing to incorporation of DOX molecules in the core of micelles compared to the empty micelles. At higher pH above 8, both micelles were in a compact, collapsed form with the *D*_h_s remained almost constant because the PDEA segments were deprotonated. And the zeta potentials at higher pH (like pH 10) were negative with increasing OH^−^ in the solution. As the pH values were ranging from 8 to 4, both micelles exhibited the gradually stretched conformation with significant increase of *D*_h_s and zeta potentials due to gradual protonation of DEA block and the increasing hydrophilicity of PDEA. At pH < 4, the *D*_h_s and zeta potentials of both micelle solutions showed sharp decrease, indicating that the PDEA segments were fully protonated with imparting a hydrophilic characteristic and the extremely strong electrostatic repulsion between polymer chains, which might cause the decrease of the aggregation number of the polymers or even slight dissociation of the micelle structures [29].

### *In vitro* drug release profiles and cell experiments

**Figure 7:**
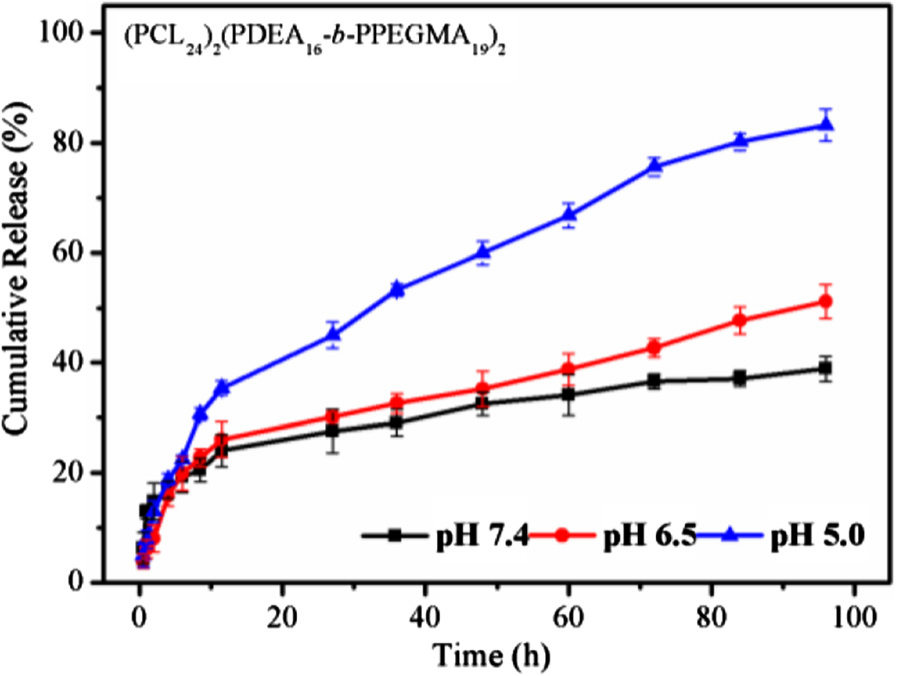
***In vitro *** **drug release profiles of DOX-loaded micelles at pH 7.4, 6.5, and 5.0.**

The *in vitro* drug release profiles of DOX-loaded micelles were evaluated at 37°C under different pH (pH 7.4, pH 6.5, and pH 5.0) to explore the effects of pH-responsive behavior on controlled drug delivery, as shown in Figure 7. The release rates significantly accelerated as the pH decreased from 7.4 to 5.0, which demonstrated that the pH of medium had a strong effect on the DOX release from the (PCL)_2_(PDEA-*b*-PPEGMA)_2_ micelles. At pH 7.4, only 22% of DOX was released in 12 h and then the release rate was almost constant, and approximately 36% of DOX was released after 96 h. At pH 6.5, the release rates of DOX accelerated to a certain extent with about 50% of DOX was released after 96 h, due to the partial protonation of the tertiary amine groups of DEA contributed to the slight swell of micelles. At pH 5.0, as the most of the tertiary amine groups of DEA had been protonated, the distinctly decreased hydrophobicity of the micellar core and greatly increased electrostatic repulsion between DEA moieties contributed to the greater degree of swell or even slight dissociation of micelles, the release rates of DOX were drastically accelerated, the cumulative release of DOX was 40% in 12 h, 60% in 48 h, and almost 82% in 96 h. Moreover, initial burst drug release was not observed.

To deeply apprehend the pH-triggered hydrophobic drug release behavior, a semi-empirical equation (1) established by Siepmann and Peppas [46] is considered to analyze the drug release mechanism from the micelles by fitting these kinetic data for the onset stage of release [42,47].

(1) $$\log\left(\frac{M_{t}}{M_{\infty }}\right)=n\log t+\log k$$

Where *M*_t_ and *M*_∞_ are the absolute cumulative amount of drug released at time *t* and infinite time respectively, *n* is the release exponent indicating the drug release mechanism and *k* is a constant incorporating structural and geometric characteristic of the device. For spherical particles, the value of *n* is equal to 0.43 for Fickian diffusion and 0.85 for non-Fickian mechanism, *n* < 0.43 is due to the combination of diffusion and erosion control, and 0.43 < *n* < 0.85 corresponds to anomalous transport mechanism [48].

The fitting parameters, including the release exponent *n*, rate constant *k*, and the correlation coefficient *R*^2^, were shown in Additional file 1: Table S1. The release of DOX at different pH conditions were divided into two stages with good linearity, one is from 0 to 12 h, and the other is from 12 to 96 h. The results showed that the pH values have major influence on DOX release process. In the first 12 h, the *n* values of pH 7.4, 6.5, and 5.0 were 0.28, 0.49, and 0.63, respectively. The drug release rates were significantly accelerated and the mechanism of DOX transformed from the combination of diffusion and erosion control to anomalous transport mechanism action when changing pH from 7.4 to 5.0. After 12 h, drug release was controlled by anomalous transport mechanism action with the *n* values of pH 7.4, 6.5, and 5.0 were 0.48, 0.49, and 0.50, respectively.

**Figure 8:**
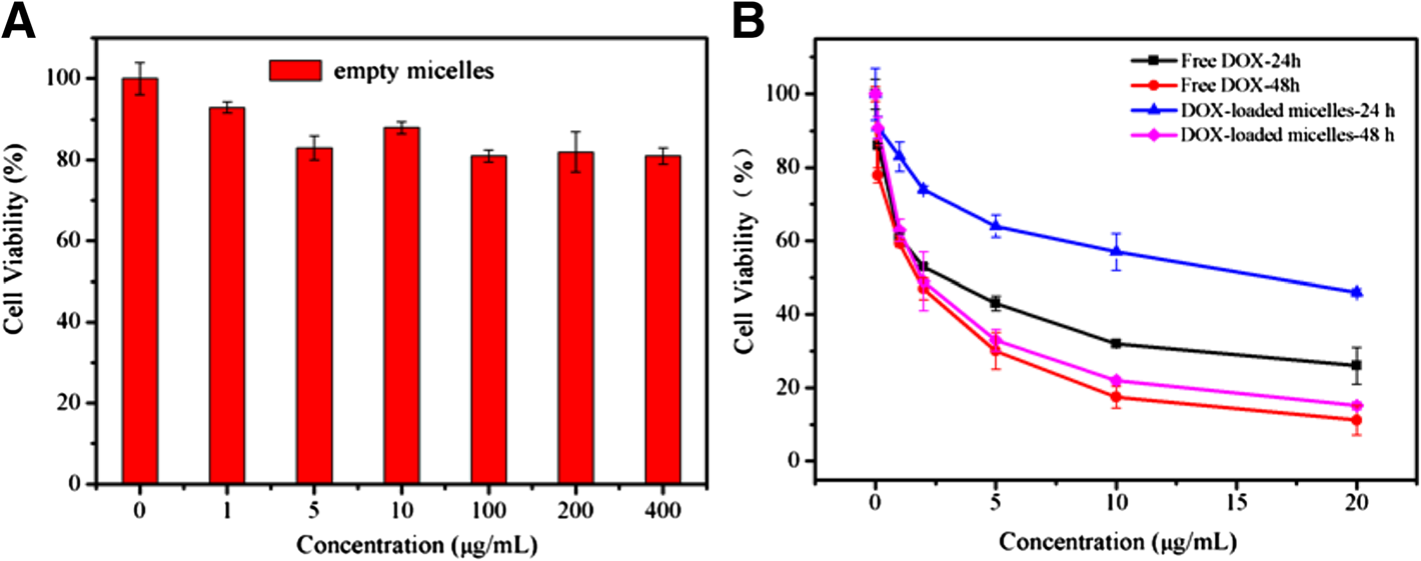
***In vitro *cytotoxicity.** Empty micelles after 48 h. At different concentrations of polymer **(A)** and DOX-loaded micelles after 24 h and 48 h **(B)** incubation at different concentrations of DOX determined by MTT assay against HepG2 cells. The standard deviation for each data point was averaged three samples (*n* = 3).

**Figure 9:**
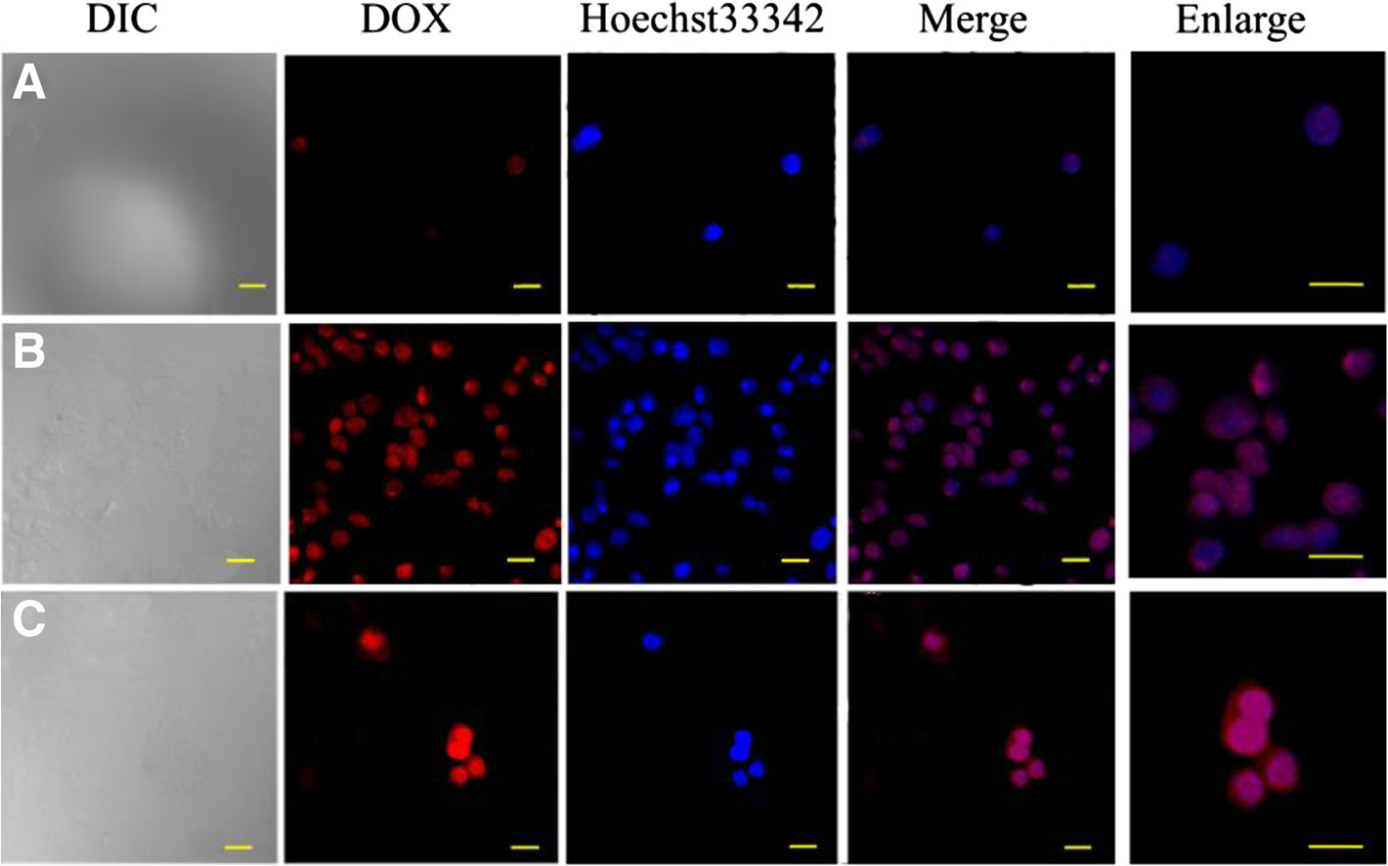
**CLSM images of HepG2 cells.** For incubation with DOX-loaded micelles. For 4 h **(A)**, 24 h **(B)**, and with free DOX for **(C)** 24 h (red, DOX; blue, Hoechst 33324. Scale bar, 20 μm).

The cytotoxicity of free DOX, empty micelles and DOX-loaded micelles against HepG2 (hepatocellular carcinoma) cells were determined by MTT assay [8,49,50]. It should be noted that the empty micelles exhibited negligible cytotoxicity, as about 80% viability was observed even at their highest concentration (400 μg/mL) after 48 h incubation in Figure 8A. Figure 8B showed the viability of HepG2 cells in the presence of free DOX and DOX-loaded micelles. The IC_50_ values were 1.6 and 2.0 μg/mL for free DOX and DOX-loaded micelles after 48 h incubation, respectively, indicating that the DOX-loaded micelles could exhibit similar antitumor activities to free DOX. Compared with free DOX, DOX-loaded micelles exhibited much lower cytotoxicity to HepG2 cells at the same dose of DOX, which was mostly due to the controlled and incomplete release of DOX from micelles in this time frame, as confirmed with *in vitro* DOX release.The cellular uptake of the micelles was further examined by CLSM measurements. HepG2 cells were cultured with free DOX and DOX-loaded micelles (50 μg/mL of DOX concentration) at 37°C for 4 and 24 h, respectively. The red fluorescence was mainly observed in cytoplasm with a small portion in the nuclei after 4 h (Figure 9A). With further incubation for 24 h in Figure 9B, intense DOX red fluorescence was almost localized in the nuclei, but not so strong as that of free DOX (Figure 9C), indicating that DOX-loaded micelles might not enter the nuclei as quickly as the free DOX. Because DOX is a small molecule, it can be quickly transported into cells and enter the nuclei through a passive diffusion mechanism. However, DOX-loaded micelles are internalized through an endocytotic pathway and only the released DOX can enter nuclei.

## Conclusions

Serial amphiphilic miktoarm star polymers (PCL)_2_(PDEAEMA-*b*-PPEGMA)_2_ were successfully prepared by a combination of ROP and continuous ARGET ATRP. A good first-order kinetic characteristic was observed for the continuous ARGET ATRP of DEA and PEGMA. The CMC values of (PCL)_2_(PDEA-*b*-PPEGMA)_2_ were extremely low (0.0024 to 0.0043 mg/mL). The self-assembled empty and DOX-loaded micelles were spherical in morphologies with average sizes of 63 and 110 nm depending on the architecture of the copolymers. The pH responsiveness and *in vitro* release properties from the micelles exhibited desired pH dependence owing to the protonation of tertiary amine groups of DEA. The *in vitro* release study showed that the release of DOX at pH 5.0 was much faster than that at pH 7.4 and pH 6.5. Moreover, *in vitro* cytotoxicity of DOX-loaded micelles suggested that they could effectively inhibit the growth of cancer cells HepG2 with IC_50_ of 2.0 μg/mL, indicating that the DOX-loaded (PCL)_2_(PDEA-*b*-PPEGMA)_2_ micelles could exhibit similar antitumor activities to free DOX. Intracellular uptake demonstrated that DOX was delivered into the cells effectively after the cells were incubated with DOX-loaded micelles. The characteristics demonstrated that these pH-sensitive (PCL)_2_(PDEA-*b*-PPEGMA)_2_ micelles would be efficient and hopeful platforms for cancer therapy.

## Competing interests

The authors declare that they have no competing interests.

## Authors' contributions

WJL and SYN carried out all the experiments and drafted the manuscript. DX carried out the MTT assay and contributed to the revision of the manuscript. XDG, JFW, and LJZ received the study, guided its design, the interpretation of the results, and revision of the manuscript. All authors read and approved the final manuscript.

## Authors' information

WJL and DX are doctoral candidates, SYN is a master student. JFW is a professor in the School of Bioscience & Bioengineering, South China University of Technology, Guangzhou, People’s Republic of China. XDG is an assistant professor, and LJZ is a professor in the School of Chemistry and Chemical Engineering, South China University of Technology, Guangzhou, People's Republic of China.

## Supplementary Material

Additional file 1 **Characterization of (PCL)**_**2**_**(PDEA-*b*-PPEGMA)**_**2**_** micelles. Figure S1.**^1^H NMR spectrum of (OH)_2_-Br_2_ in *d*_6_-DMSO. **Figure S2.** GPC traces of (PCL_24_)_2_-Br_2_ and (PCL_24_)_2_(PDEA_16_-*b*-PPEGMA_19_)_2_. **Figure S3.** Fluorescence emission spectra of pyrene with increasing concentration of (PCL)_2_-(PDEA-*b*-PPEGMA)_2_. **Table S1.** Fitting parameters of DOX release data from DOX-loaded micelles at pH 7.4, 6.5 and 5.0. These materials are available from the Springer Library or from the author. Click here for file

## References

[B1] Husseini GA Pitt WG Micelles and nanoparticles for ultrasonic drug and gene delivery Adv Drug Del Rev 2008 9 1137 1152 10.1016/j.addr.2008.03.008 PMC2490710 18486269

[B2] Ge Z Liu S Functional block copolymer assemblies responsive to tumor and intracellular microenvironments for site-specific drug delivery and enhanced imaging performance Chem Soc Rev 2013 9 7289 7325 10.1039/c3cs60048c 23549663

[B3] Lee ES Gao Z Bae YH Recent progress in tumor pH targeting nanotechnology J Controlled Release 2008 9 164 170 10.1016/j.jconrel.2008.05.003 PMC2695946 18571265

[B4] Yang YQ Guo XD Lin WJ Zhang LJ Zhang CY Qian Y Amphiphilic copolymer brush with random pH-sensitive/hydrophobic structure: synthesis and self-assembled micelles for sustained drug delivery Soft Matter 2012 9 454 464 10.1039/c1sm06314f

[B5] Xiong XB Binkhathlan Z Molavi O Lavasanifar A Amphiphilic block co-polymers: preparation and application in nanodrug and gene delivery Acta Biomater 2012 9 2017 2033 10.1016/j.actbio.2012.03.006 22406912

[B6] Yang YQ Lin WJ Zhao B Wen XF Guo XD Zhang LJ Synthesis and physicochemical characterization of amphiphilic triblock copolymer brush containing pH-sensitive linkage for oral drug delivery Langmuir 2012 9 8251 8259 10.1021/la301099q 22568600

[B7] Tang RP Ji WH Panus D Palumbo RN Wang C Block copolymer micelles with acid-labile ortho ester side-chains: synthesis, characterization, and enhanced drug delivery to human glioma cells J Controlled Release 2011 9 18 27 10.1016/j.jconrel.2010.12.005 PMC3081958 21194551

[B8] Cui F Li Y Zhou SF Jia MM Yang XR Yu F Ye SF Hou ZQ Xie LY Chang PC A comparative in vitro evaluation of self-assembled PTX-PLA and PTX-MPEG-PLA nanoparticles Nanoscale Res Lett 2013 9 301 10.1186/1556-276X-8-301 23806106 PMC3728229

[B9] Roy D Cambre JN Sumerlin BS Future perspectives and recent advances in stimuli-responsive materials Prog Polym Sci 2010 9 278 301 10.1016/j.progpolymsci.2009.10.008

[B10] Zhuang J Gordon MR Ventura J Li L Thayumanavan S Multi-stimuli responsive macromolecules and their assemblies Chem Soc Rev 2013 9 421 7435 10.1039/c3cs60094g PMC3740153 23765263

[B11] Kelley EG Albert JNL Sullivan MO Epps TH III Stimuli-responsive copolymer solution and surface assemblies for biomedical applications Chem Soc Rev 2013 9 7057 7071 10.1039/c3cs35512h 23403471 PMC3703495

[B12] Hu J Zhang G Liu S Enzyme-responsive polymeric assemblies, nanoparticles and hydrogels Chem Soc Rev 2012 9 5933 5949 10.1039/c2cs35103j 22695880

[B13] Wei H Zhuo RX Zhang XZ Design and development of polymeric micelles with cleavable links for intracellular drug delivery Prog Polym Sci 2013 9 503 535 10.1016/j.progpolymsci.2012.07.002

[B14] Hoffmeister CRD Durli TL Schaffazick SR Raffin RP Bender EA Beck RCR Pohlmann AR Guterres SS Hydrogels containing redispersible spray-dried melatonin-loaded nanocapsules: a formulation for transdermal-controlled delivery Nanoscale Res Lett 2012 9 251 10.1186/1556-276X-7-251 22587614 PMC3463463

[B15] Lim EK Sajomsang W Choi Y Jang E Lee H Kang B Kim E Haam S Suh JS Chung SJ Huh YM Chitosan-based intelligent theragnosis nanocomposites enable pH-sensitive drug release with MR-guided imaging for cancer therapy Nanoscale Res Lett 2013 9 467 10.1186/1556-276X-8-467 24206754 PMC4226245

[B16] Shen Y Zhan Y Tang J Xu P Johnson PA Radosz M Van Kirk EA Murdoch WJ Multifunctioning pH-responsive nanoparticles from hierarchical self-assembly of polymer brush for cancer drug delivery AIChE J 2008 9 2979 2989 10.1002/aic.11600

[B17] Yu H Zou Y Wang Y Huang X Huang G Sumer BD Boothman DA Gao J Overcoming endosomal barrier by amphotericin B-loaded dual pH-responsive PDMA-b-PDPA micelleplexes for siRNA delivery ACS Nano 2011 9 9246 9255 10.1021/nn203503h 22011045 PMC4797624

[B18] Wang H Xu F Wang Y Liu X Jin Q Ji J pH-responsive and biodegradable polymeric micelles based on poly(β-amino ester)-graft-phosphorylcholine for doxorubicin delivery Polym Chem 2013 9 3012 3019 10.1039/c3py00139c

[B19] Liu H Li C Liu H Liu S pH-responsive supramolecular self-assembly of well-defined zwitterionic ABC miktoarm star terpolymers Langmuir 2009 9 4724 4734 10.1021/la803813r 19239225

[B20] Wang Y Grayson SM Approaches for the preparation of non-linear amphiphilic polymers and their applications to drug delivery Adv Drug Del Rev 2012 9 852 865 10.1016/j.addr.2012.03.011 22465560

[B21] Khanna K Varshney S Kakkar A Miktoarm star polymers: advances in synthesis, self-assembly, and applications Polym Chem 2010 9 1171 1185 10.1039/c0py00082e

[B22] Cho HY Averick SE Paredes E Wegner K Averick A Jurga S Das SR Matyjaszewski K Star polymers with a cationic core prepared by ATRP for cellular nucleic acids delivery Biomacromolecules 2013 9 1262 1267 10.1021/bm4003199 23560989

[B23] Tang XL Cai SY Zhang RB Liu P Chen HB Zheng Y Sun LL Paclitaxel-loaded nanoparticles of star-shaped cholic acid-core PLA-TPGS copolymer for breast cancer treatment Nanoscale Res Lett 2013 9 420 10.1186/1556-276X-8-420 24134303 PMC3874754

[B24] Wang D Chen H Su Y Qiu F Zhu L Huan X Zhu B Yan D Guo F Zhu X Supramolecular amphiphilic multiarm hyperbranched copolymer: synthesis, self-assembly and drug delivery applications Polym Chem 2013 9 85 94 10.1039/c2py20573d

[B25] Chen B van der Poll DG Jerger K Floyd WC Fréchet JMJ Szoka FC Synthesis and properties of star-comb polymers and their doxorubicin conjugates Bioconjugate Chem 2011 9 617 624 10.1021/bc100400u PMC3101114 21375296

[B26] He E Ravi P Tam KC Synthesis and self-assembly behavior of four-arm poly(ethylene oxide)-b-poly(2-(diethylamino)ethyl methacrylate) star block copolymer in salt solutions Langmuir 2007 9 2382 2388 10.1021/la062987+ 17261049

[B27] He E Yue CY Simeon F Zhou LH Too HP Tam KC Polyplex formation between four-arm poly(ethylene oxide)-b-poly(2-(diethylamino)ethyl methacrylate) and plasmid DNA in gene delivery J Biomed Mater Res Part A 2009 9 708 718 10.1002/jbm.a.32255 19048636

[B28] Knop K Pavlov GM Rudolph T Martin K Pretzel D Jahn BO Scharf DH Brakhage AA Makarov V Mollmann U Schacher FH Schubert US Amphiphilic star-shaped block copolymers as unimolecular drug delivery systems: investigations using a novel fungicide Soft Matter 2013 9 715 726 10.1039/c2sm26509e

[B29] Yang YQ Zhao B Li ZD Lin WJ Zhang CY Guo XD Wang JF Zhang LJ pH-sensitive micelles self-assembled from multi-arm star triblock co-polymers poly(ϵ-caprolactone)-b-poly(2-(diethylamino)ethyl methacrylate)-b-poly(poly (ethylene glycol) methyl ether methacrylate) for controlled anticancer drug delivery Acta Biomater 2013 9 7679 7690 10.1016/j.actbio.2013.05.006 23669619

[B30] Gou PF Zhu WP Shen ZQ Calixarene-centered amphiphilic A_2_B_2_ miktoarm star copolymers based on poly(ϵ-caprolactone) and poly(ethylene glycol): synthesis and self-assembly behaviors in water J Polym Sci Part A: Polym Chem 2010 9 5643 5651 10.1002/pola.24316 20225892

[B31] Zhang W Zhang W Cheng Z Zhou N Zhu J Zhang Z Chen G Zhu X Synthesis and aggregation behaviors of nonlinear multiresponsive, multihydrophilic block copolymers Macromolecules 2011 9 3366 3373 10.1021/ma200083v

[B32] Wolf FF Friedemann N Frey H Poly(lactide)-block-poly(HEMA) block copolymers: an orthogonal one-pot combination of ROP and ATRP, using a bifunctional initiator Macromolecules 2009 9 5622 5628 10.1021/ma900894d

[B33] Cai T Yang WJ Neoh KG Kang ET Preparation of jellyfish-shaped amphiphilic block-graft copolymers consisting of a poly(ϵ-caprolactone)-block- poly(pentafluorostyrene) ring and poly(ethylene glycol) lateral brushes Polym Chem 2012 9 1061 1068 10.1039/c2py00609j

[B34] Matyjaszewski K Jakubowski W Min K Tang W Huang J Braunecker WA Tsarevsky NV Diminishing catalyst concentration in atom transfer radical polymerization with reducing agents PNAS 2006 9 15309 15314 10.1073/pnas.0602675103 17032773 PMC1622823

[B35] Nicolaÿ R Kwak Y Matyjaszewski K A green route to well-defined high-molecular-weight (co)polymers using ARGET ATRP with alkyl pseudohalides and copper catalysis Angew Chem, Int Ed 2010 9 551 554 10.1002/ange.200905340 20013835

[B36] Pasquale AJ Long TE Real-time mnitoring of the stable free radical polymerization of styrene via in-situ mid-infrared spectroscopy Macromolecules 1999 9 7954 7957 10.1021/ma9912498

[B37] Pasquale AJ Long TE Synthesis of star-shaped polystyrenes via nitroxide-mediated stable free-radical polymerization J Polym Sci Part A: Polym Chem 2001 9 216 223 10.1002/1099-0518(20010101)39:1<216::AID-POLA240>3.0.CO;2-Z

[B38] Zhang W Zhang W Zhou N Zhu J Cheng Z Zhu X Synthesis of miktoarm star amphiphilic block copolymers via combination of NMRP and ATRP and investigation on self-assembly behaviors J Polym Sci Part A: Polym Chem 2009 9 6304 6315 10.1002/pola.23673

[B39] Xu J Ge Z Zhu Z Luo S Liu H Liu S Synthesis and micellization properties of double hydrophilic A_2_BA_2_ and A_4_BA_4_ non-linear block copolymers Macromolecules 2006 9 8178 8185 10.1021/ma061934w

[B40] Zhang L Guo R Yang M Jiang X Liu B Thermo and pH dual-responsive nanoparticles for anti-cancer drug delivery Adv Mater 2007 9 2988 2992 10.1002/adma.200601817

[B41] Yang YQ Zheng LS Guo XD Qian Y Zhang LJ pH-sensitive micelles self-assembled from amphiphilic copolymer brush for delivery of poorly water-soluble drugs Biomacromolecules 2010 9 116 122 21121600 10.1021/bm101058w

[B42] Zhang HW Cai GQ Tang GP Wang LQ Jiang HL Synthesis, self-assembly, and cytotoxicity of well-defined trimethylated chitosan-O-poly(ϵ-caprolactone): effect of chitosan molecular weight J Biomed Mater Res Part B 2011 9 290 299 10.1002/jbm.b.31851 21604366

[B43] Lele BS Leroux JC Synthesis and micellar characterization of novel amphiphilic A-B-A triblock copolymers of N-(2-hydroxypropyl)methacrylamide or N-vinyl-2-pyrrolidone with poly(ϵ-caprolactone) Macromolecules 2002 9 6714 6723 10.1021/ma020433h

[B44] Guo XD Tandiono F Wiradharma N Khor D Tan CG Khan M Qian Y Yang YY Cationic micelles self-assembled from cholesterol-conjugated oligopeptides as an efficient gene delivery vector Biomaterials 2008 9 4838 4846 10.1016/j.biomaterials.2008.07.053 18829102

[B45] Guo XD Zhang LJ Chen Y Qian Y Core/shell pH-sensitive micelles self-assembled from cholesterol conjugated oligopeptides for anticancer drug delivery AIChE J 2010 9 1922 1931

[B46] Siepmann J Peppas NA Modeling of drug release from delivery systems based on hydroxypropyl methylcellulose (HPMC) Adv Drug Del Rev 2012 9 Supplement 163 174 10.1016/s0169-409x(01)00112-0 11369079

[B47] Siepmann J Göpferich A Mathematical modeling of bioerodible, polymeric drug delivery systems Adv Drug Del Rev 2001 9 229 247 10.1016/S0169-409X(01)00116-8 11369084

[B48] Liu Y Chen Z Liu C Yu D Lu Z Zhang N Gadolinium-loaded polymeric nanoparticles modified with anti-VEGF as multifunctional MRI contrast agents for the diagnosis of liver cancer Biomaterials 2011 9 5167 5176 10.1016/j.biomaterials.2011.03.077 21521627

[B49] Wang H Xu F Li D Liu X Jin Q Ji J Bioinspired phospholipid polymer prodrug as a pH-responsive drug delivery system for cancer therapy Polym Chem 2013 9 2004 2010 10.1039/c2py20981k

[B50] Liu G Jin Q Liu X Lv L Chen C Ji J Biocompatible vesicles based on PEO-b-PMPC/[α]-cyclodextrin inclusion complexes for drug delivery Soft Matter 2011 9 662 669 10.1039/c0sm00708k

