# Supplementary material for: pH-responsive micelles based on (PCL)2(PDEA-b-PPEGMA)2 miktoarm polymer: controlled synthesis, characterization, and application as anticancer drug carrier
Source: Nanoscale Res Lett. 2014 May 18;9(1):243. doi: 10.1186/1556-276X-9-243 (PMC4046072; doi:10.1186/1556-276X-9-243)
Supplement: Additional file 1 — Characterization of (PCL) 2 (PDEA- b -PPEGMA) 2 micelles. Figure S1.1H NMR spectrum of (OH)2-Br2 in d6-DMSO. Figure S2. GPC traces of (PCL24)2-Br2 and (PCL24)2(PDEA16-b-PPEGMA19)2. Figure S3. Fluorescence emission spectra of pyrene with increasing concentration of (PCL)2-(PDEA-b-PPEGMA)2. Table S1. Fitting parameters of DOX release data from DOX-loaded micelles at pH 7.4, 6.5 and 5.0. These materials are available from the Springer Library or from the author. [file 1556-276X-9-243-S1.pdf]

Additional file

pH-Responsive Micelles Based on $(PCL)_2(PDEA$-*b*-$PPEGMA)_2$ Miktoarm Polymer: Controlled Synthesis, Characterization and Application as Anticancer Drug Carrier

Wenjing Lin,[1] Shuyu Nie,[1] Di Xiong,[1] Xindong Guo,[1] Jufang Wang,[2] Lijuan Zhang[1,*]

[1] School of Chemistry and Chemical Engineering, South China University of Technology, Guangzhou 510640, P R China

[2] School of Bioscience & Bioengineering, South China University of Technology, Guangzhou 510640, P R China

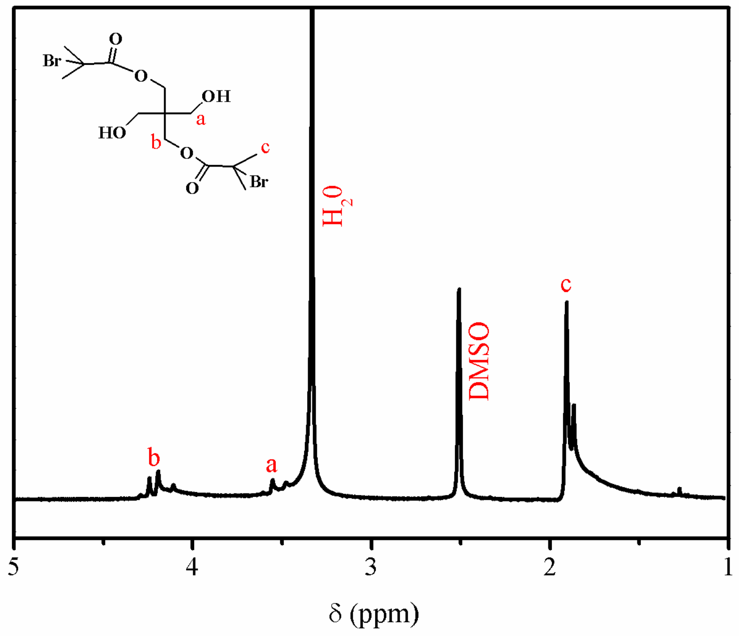

Figure S1 ^{1}H NMR spectrum of $(OH)_2$-Br_2 in d_6-DMSO.

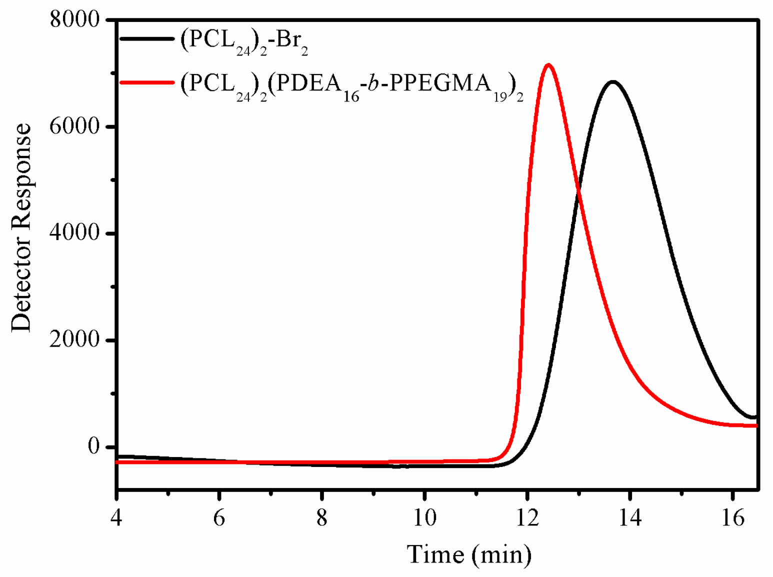

Figure S2 GPC traces of $(PCL_{24})_2$-Br_2 and $(PCL_{24})_2(PDEA_{16}$-*b*-$PPEGMA_{19})_2$.

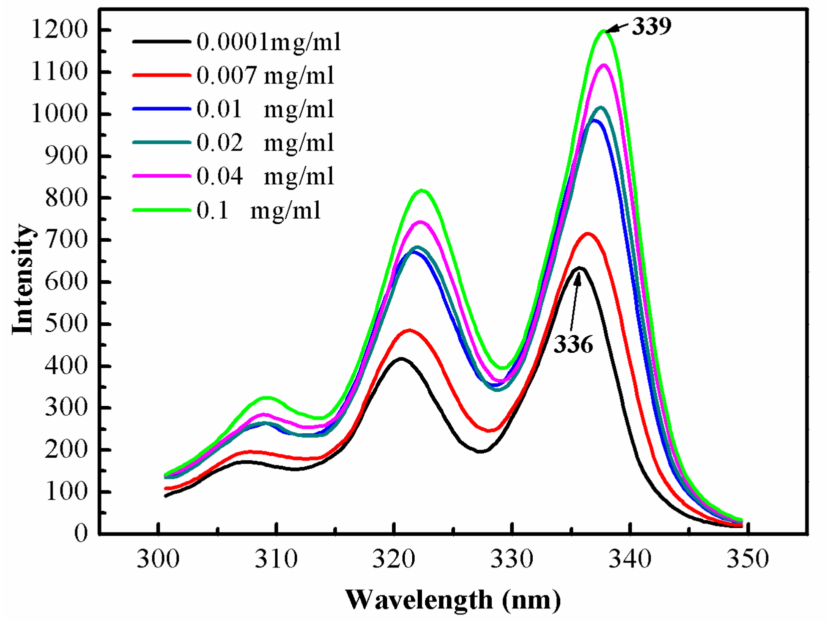

Figure S3 Fluorescence emission spectra of pyrene with increasing concertration of $(PCL)_2$-(PDEA-*b*-$PPEGMA)_2$.

Table S1 Fitting parameters of DOX release data from DOX-loaded micelles at pH 7.4, 6.5 and 5.0

pH	n_1[a]	k_1[a]	R^2_1	n_2[b]	k_2[b]	R^2_2
7.4	0.28	0.07	0.968	0.48	0.03	0.931
6.5	0.49	0.05	0.969	0.49	0.04	0.990
5.0	0.63	0.08	0.995	0.50	0.09	0.994

[a] The first stage is 0-12 h.
[b] The second stage is 12-96 h.
